# Adenosine A_2A _receptors promote collagen production by a Fli1- and CTGF-mediated mechanism

**DOI:** 10.1186/ar4229

**Published:** 2013-05-11

**Authors:** Edwin SL Chan, Hailing Liu, Patricia Fernandez, Alex Luna, Miguel Perez-Aso, Andreea M Bujor, Maria Trojanowska, Bruce N Cronstein

**Affiliations:** 1 Department of Medicine, New York University School of Medicine, 550 First Ave., New York, NY 10016, USA; 2 Laboratory of Receptor Biology and Gene Expression, National Cancer Institute, NIH, Bethesda, MD 20892, USA; 3 Department of Medicine, Arthritis Center, Boston University School of Medicine, 72 East Concord Street, Boston, MA 02118, USA

**Keywords:** Fibrosis, fibroblast, scleroderma

## Abstract

**Introduction:**

Adenosine, acting through the A_2A _receptor, promotes tissue matrix production in the skin and the liver and induces the development of dermal fibrosis and cirrhosis in murine models. Since expression of A_2A _receptors is increased in scleroderma fibroblasts, we examined the mechanisms by which the A_2A _receptor produces its fibrogenic effects.

**Methods:**

The effects of A_2A _receptor ligation on the expression of the transcription factor, Fli1, a constitutive repressor for the synthesis of matrix proteins, such as collagen, is studied in dermal fibroblasts. Fli1 is also known to repress the transcription of CTGF/CCN2, and the effects of A_2A _receptor stimulation on CTGF and TGF-β1 expression are also examined.

**Results:**

A_2A _receptor occupancy suppresses the expression of Fli1 by dermal fibroblasts. A_2A _receptor activation induces the secretion of CTGF by dermal fibroblasts, and neutralization of CTGF abrogates the A_2A _receptor-mediated enhancement of collagen type I production. A_2A_R activation, however, resulted in a decrease in TGF-β1 protein release.

**Conclusions:**

Our results suggest that Fli1 and CTGF are important mediators of the fibrogenic actions of adenosine and the use of small molecules such as adenosine A_2A _receptor antagonists may be useful in the therapy of dermal fibrosis in diseases such as scleroderma.

## Introduction

Matrix production is critical for wound healing and tissue repair but in some clinical settings overproduction of matrix provides the basis for organ injury and dysfunction [1]. In previous studies, we have demonstrated that occupancy of adenosine A_2A _receptors by an appropriate agonist, such as CGS-21680, promotes more rapid wound closure in both normal mice and diabetic rats and the enhancement in dermal wound healing is accompanied by an increase in matrix (collagen) in the wounds [2-4]. We have recently shown that pharmacological A_2A_R blockade can be used to diminish scarring while improving the collagen composition and tensile strength of the healed wound [5]. Adenosine A_2A _receptor agonists such as CGS21680 do not promote wound healing in mice lacking adenosine A_2A _receptors and these same A_2A _receptor-deficient mice are protected from the development of hepatic and dermal fibrosis as well [6,7]. Although exogenous adenosine receptor agonists may promote wound healing, endogenous adenosine production also contributes to wound healing and pathologic fibrosis because mice lacking adenosine A_2A _receptors have prolonged wound healing and mice that underproduce adenosine in response to appropriate stimuli due to deletion of ecto-5'nucleotidase are protected from the development of hepatic fibrosis [4,8]. Consistent with the role of matrix overproduction in organ fibrosis and dysfunction, we have further demonstrated that adenosine and its receptor play a critical role in models of hepatic fibrosis/cirrhosis and dermal fibrosis in a model of scleroderma [6,7,9] and others have reported that adenosine plays a role in postoperative abdominal adhesions [10].

Although adenosine and adenosine A_2A _receptors regulate the function of many cell types that stimulate matrix production and fibrosis indirectly, we have reported that stimulation of the A_2A _receptor on matrix producing fibroblasts in the skin and stellate cells in the liver directly stimulates collagen production [6,7]. In a murine model deficient for adenosine deaminase, a key catabolizing enzyme for adenosine, we have shown that fibrosis developed spontaneously in the skin in the absence of exogenous fibrogenic stimulators, concomitant with an increase in adenosine levels in the skin [9]. In primary human dermal fibroblasts, the collagen-inducing effect of adenosine can be attributed directly to A_2A _receptor occupancy, since it is not attenuated by A_1 _receptor or A_2B _receptor antagonism, and A_3 _receptors are not expressed on human dermal fibroblasts [6]. Furthermore, A_2A _receptor-deficient mice or mice treated with an A_2A _receptor antagonist are protected against induction of fibrogenesis in the skin by administration of bleomycin [6]. A_2A _receptor antagonism also diminished dermal fibrogenesis in adenosine deaminase-deficient mice [9]. Lazzerini *et al*. have shown that expression of adenosine A_2A _receptors are increased three-fold in dermal fibroblasts isolated from scleroderma patients compared to non-diseased controls, and A_2A _receptor ligation promotes collagen synthesis in dermal fibroblasts [11]. The adenosine A_2A _receptor is a G_S_-linked receptor that stimulates cAMP accumulation and other downstream signals that mediate activation of collagen production by fibroblasts and stellate cells. A number of downstream signals play a role in the stimulation of collagen production by stellate cells including activation of the downstream kinases ERK1/2 (linked to collagen type I production) and p38 MAPKinase (linked to collagen type III production) [12].

Matrix production depends on the balance between forces that promote the transcription of matrix-producing genes and those that repress them. One of the best characterized of such repressors to date is friend leukemia integration-1 (Fli1), a member of the E26 transformation-specific (Ets) transcription factor family [13-16]. Reduction of Fli1 levels in dermal fibroblasts is associated with increased synthesis of connective tissue growth factor (CTGF/CCN2) and type I collagen, as well as a reduction of MMP-1 production [15-19]. This bears much similarity to the well known profibrotic effects of TGF-β. Indeed, enforced expression of Fli1 almost entirely abrogated TGF-β-induced profibrotic gene expression [16], suggesting that Fli1 is a potent endogenous antagonist of TGF-β signaling. Asano and colleagues have recently shown that TGF-β elicits PCAF-dependent acetylation of Fli1, which in turn results in its dissociation from the collagen promoter and subsequent degradation [20,21]. We therefore hypothesized that one of the possible mechanisms for the fibrogenic effects of adenosine is mediated through suppression of Fli1 expression. Furthermore, since CTGF is a matrix-promoting growth factor regulated by Fli1 and is vastly overexpressed in adenosine deaminase-deficient mice [9,19], it may also be an important regulator of A_2A _receptor-mediated fibrous tissue production.

## Materials and methods

### Reagents

CGS-21680 was purchased from Sigma (St. Louis, MO, USA) and ZM-241385 was from Tocris (Ballwin, MO, USA). Antibody for human Fli1 was from BD Biosciences (San Jose, CA, USA), anti-CTGF and anti β-actin were from Santa Cruz Biotechnology (Santa Cruz, CA, USA), anti-human p84 was from Abcam (Cambridge, MA, USA) and anti-collagen I was purchased from Southern Biotech (Birmingham, AL, USA). Anti-mouse and anti-rabbit immunoglobulins were from DAKO (Carpinteria, CA, USA). DMEM and cell culture reagents were from Invitrogen (Grand Island, NY, USA).

### Cell culture

Primary adult human dermal fibroblasts were purchased from Cambrex (East Rutherford, NJ, USA), or Lonza, Inc. (Allendale, NJ, USA). Fibroblasts were cultured in DMEM and used for experiments from first to the fifth passages. For CTGF neutralization, the human dermal fibroblasts where harvested during 16 hours and then incubated with the CTGF antibody (1:250) or normal serum as a control.

### Western blotting

To examine expression of Fli1 protein, nuclear extracts from treated dermal fibroblasts were prepared following manufacturer´s instructions (Active Motif Nuclear Extract Protocol, Panomics, Fremont, CA, USA). Protein concentration was measured by BCA method (Pierce, Rockford, IL, USA) using bovine serum albumin as standard. Nuclear extracts (30 µg/lane) were electrophoresed in 12.5% SDS-polyacrylamide gel and transferred onto a nitrocellulose membrane (Bio-Rad, Hercules, CA, USA). The nitrocellulose membrane was blocked for two hours at room temperature in blocking solution (3% non-fat dry powdered milk in 1x Tween 20 Tris buffered saline (TTBS consisting of 20 mM Tris-HCl, pH 7.4, 150 mM NaCl and 0.1% Tween 20). After blocking, the membrane was incubated with primary antibody Fli1 (1:500; BD Pharmagen, San Jose, CA, USA), p84 (1:1,000; Abcam, Cambridge, MA, USA) or β-Actin (1:1,000; Santa Cruz Biotechnology Inc.,) overnight at 4ºC with gentle shaking on a platform shaker. After incubation with secondary antibody (rabbit anti-mouse immunoglobulins, 1:1,000 dilution, one hour, room temperature), proteins were visualized using the ECF kit (Amersham Biosciences, Little Chalfont, Buckinghamshire, UK). Band intensities were quantified by Adobe Photoshop 7.0.1 software and normalized to p84 nuclear marker level. To examine the expression of CTGF/CCN2, cells were plated 5x10^4^/cm² into six-well plates, starved overnight when cell number reached 75% confluence and supernates were collected following treatment. Samples were concentrated using Microcon Ultracel YM-35 (Millipore, **Billerica**, MA, USA), and concentrated supernates were electrophoresed in a 10% SDS-polyacrylamide gel. CTGF/CCN2 was detected with primary anti-CTGF/CCN2antibody (1:1,000, Santa Cruz Biotechnology). Collagen type I was assessed in cell lysates as previously described [6].

### Reverse transcription and Real-time PCR

Total RNA from treated primary human dermal fibroblasts was isolated using Trizol (Invitrogen, Carlsbad, CA, USA) following stimulation with CGS-21680 (10 µM) according to the manufacturer's protocol. RNA was quantified using spectrophotometric OD_260 _measurements and quality was assessed by OD_260_/ OD_280 _ratio. Reverse transcription was performed using the GeneAmp RNA Core Kit (Applied Biosystems, Carlsbad, CA, USA) in a volume of 25 µl using oligo dT primers and MuLV reverse transcriptase according to the manufacturer's protocol. Real-time PCRs were performed using the SYBR Green PCR Master Mix (Stratagene, Santa Clara, CA, USA) following the manufacturer's instructions and carried out on the Mx3005P™Q-PCR system (Stratagene). Aliquots of reverse transcription reactions were subjected to PCR in 25 µl reactions with SYBR® green using primers for Fli1 [5'- caacacggaagtgctgttgt-3' (forward), 5'-ccaaggggaggacttttgtt-3' (reverse)], CTGF/CCN2 [5'-ttccagagcagctgcaagta-3' (forward), 5'-ctcgtcacacacccactcc-3' (reverse), GAPDH [5'-accatcatccctgcctctac-3' (forward) and 5'-cctgttgctgtagccaaat-3' (reverse)], COL1A1 [5'-tgttcagctttggacctccg-3'(forward), 5'-ccgttctgtacgcaggtgattg-3' (reverse)], and COL3A1 [5'-gaagatgtccttgatgtgc-3'(forward), 5'-agccttgcgtgttcgatat-3'(reverse)].

Primers were designed using output Primer 3 software [22]. The thermal cycling conditions included an initial 95°C for 300 s; then 95°C for 60 s, 58°C for 45 s and 72°C for 45 s for 40 cycles for Fli-1 and 95°C for 300 s; then 95°C for 60 s, 57°C for 45 s and 72°C for 45 s for 40 cycles for CTGF/CCN2. For each assay, standards, no-template and no-RT controls were included to verify the quality and cDNA specificity of the primers. Comparison of the expression of each gene between its control and stimulated states was determined with the delta-delta (ΔΔ)Ct, according to the following formula: (ΔΔ)Ct = ((Ct_GOI Control _- Ct _HKG Control_) - (Ct_GOI Stim _- Ct _HKG Stim_)), where GOI (gene of interest) corresponds to Fli1, CTGF or COL1A1 and HKG (housekeeping gene) corresponds to GAPDH. Fold increase was calculated according to the formula: Fold = 2 ^(ΔΔ)Ct ^[23].

### Determination of transforming growth factor -β1

Normal human dermal fibroblasts were serum starved for 24 h and stimulated with CGS-21680 (1µM). Then, transforming growth factor β1 (TGF-β1) was determined in the supernates after removing the debris by centrifugation with the TGF-β1 ELISA kit (R&D Systems, Minneapolis, MN, USA) according to the manufacturer's protocol.

### Statistics

Results are represented as mean ± SEM. Data were analyzed by one-way ANOVA and significance of differences between groups was determined by Newman-Keuls method. Pairwise comparisons were made with the unpaired t-test. All statistical analyses were performed with Graphpad Prism software v. 4.02 (Graphpad Software, La Jolla, CA, USA).

## Results

### Adenosine A_2A _receptor activation suppresses Fli1 expression in human dermal fibroblasts

**Figure 1:**
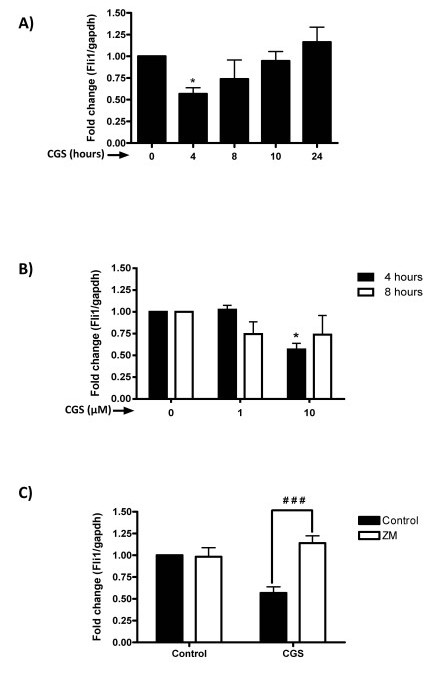
**Adenosine A_2A _receptor occupancy suppresses Fli1 mRNA expression in human dermal fibroblasts**. NHDF cells were incubated with CGS-26180 at **A) **10 µM at the indicated time-points or **B) **1 µM or 10 µM for four or eight hours. **C) **Incubation with the A_2A _receptor-selective antagonist ZM241385 (1 µM) prior to CGS-26180 (10 µM) for four hours prevents the decrease on Fli1 expression. Data represent means ± S.E.M. of four to eight independent experiments. *P <0.05 *vs*. non-stimulated control (One-way ANOVA, post-hoc analysis by Newman-Keuls test); ^###^*P *<0.001, CGS-26180 + ZM241385 *vs*. CGS-26180 (unpaired t-test).

**Figure 2:**
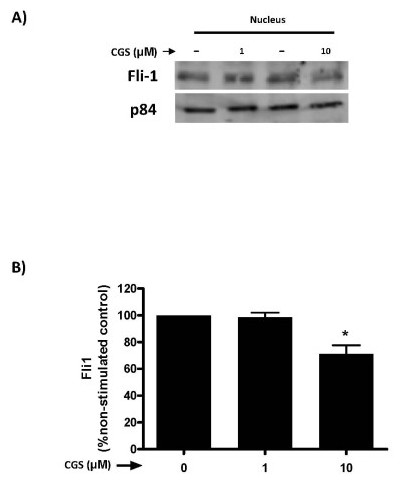
**Adenosine A_2A _receptor occupancy inhibits nuclear Fli1 protein expression in human dermal fibroblasts**. NHDF cells were incubated with CGS-26180 at 1 to 10 µM over 24 hours and cellular fractionation was performed as described under "Methods". **A) **Representative Western-blot images showing Fli1 and the nuclear marker p84. **B) **Bands were quantified and data represent means ± S.E.M. from three independent experiments. Statistics were performed by one-way ANOVA with *post-hoc *analysis by Newman-Keuls test, *P <0.05 *vs*. non-stimulated control.

We have previously demonstrated that adenosine A_2A _receptor activation increases collagen production in dermal fibroblasts, including type I and III collagens [6], although the molecular events involved are unclear. Since suppression of Fli1 expression has also been associated with increased collagen synthesis [16], we investigated whether adenosine A_2A _receptor activation results in alteration of Fli1 message expression. Following incubation with the adenosine A_2A _agonist, CGS-21680, Fli1 mRNA expression in dermal fibroblasts was reduced significantly compared to control at four hours (56.8 ± 6.9%, *n *= 8, *P *<0.05) (Figure 1A), but not at longer time-points (*P *= NS). The effect of CGS-21680 on down-regulation of Fli1 message was elicited with CGS-21680 (10 µM), (Figure 1B). While the A_2A _receptor antagonist ZM241385 did not change the basal values for Fli1 mRNA, the impact of CGS-21680 on Fli1 expression was significantly prevented by coincubation with ZM241385 (1 μM, *n *= 8, *P *<0.001 vs CGS) (Figure 1C). Similar to Fli1 message, nuclear Fli1 protein expression in the nucleus was also significantly suppressed by adenosine A_2A _receptor activation compared to control (71.3 ± 6.3% of control, *n *= 3, *P *<0.05), (Figure 2).

### Adenosine A_2A _receptor activation promotes CTGF secretion by dermal fibroblasts

**Figure 3:**
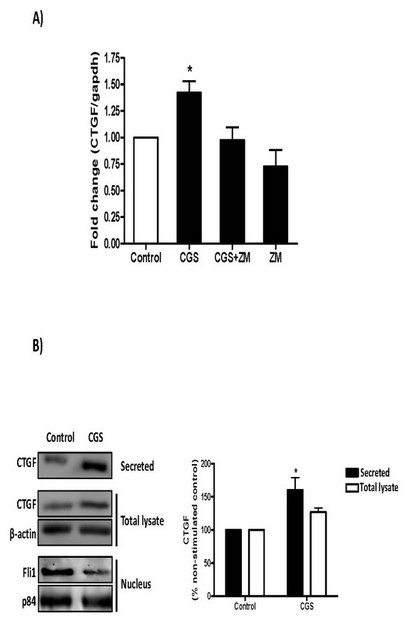
**Adenosine A_2A _receptor occupancy increases CTGF mRNA and protein expression and secretion**. NHDF cells were incubated with CGS-26180 at 1 µM **A) **for 4 hours for CTGF mRNA analysis or **B) **for 24 hours prior to cellular fractionation and protein concentration from supernates as described under "Methods". Bands were quantified and data represent means ± S.E.M. from three to eight independent experiments. Statistics were performed by one-way ANOVA with *post-hoc *analysis by Newman-Keuls test, *P <0.05 *vs*. non-stimulated control.

We have previously reported that immunohistochemical staining for CTGF (CCN2) is increased in adenosine deaminase-deficient mice which have high tissue and circulating adenosine levels compared to wild-type littermate control mice [9] and therefore sought to determine whether the effects of adenosine A_2A _receptor activation on fibroblast function is associated with enhanced CTGF production. Message for CTGF in dermal fibroblasts was increased to 1.47 ± 0.11-fold that of control following treatment with the A_2A _receptor agonist, CGS-21680 (*n *= 8, *P *<0.05 vs. control) (Figure 3A). The CGS-21680-induced increase in CTGF was reversed by pretreatment with the A_2A _antagonist ZM142385, while ZM241385 alone did not alter CTGF mRNA expression. While Fli1 nuclear protein expression was decreased, CTGF protein levels in supernates (secreted CTGF) and cell-associated CTGF (total lysate) in dermal fibroblast cultures treated with CGS-21680 were also increased (to 160.3 ± 18.7% of control, secreted CTGF, *n *= 5, *P *<0.05; and 127.0 ± 6.0% of control, total lysate, *P *= NS, respectively) (Figure 3B).

### CTGF is necessary for A_2A _receptor-mediated collagen type I production

**Figure 4:**
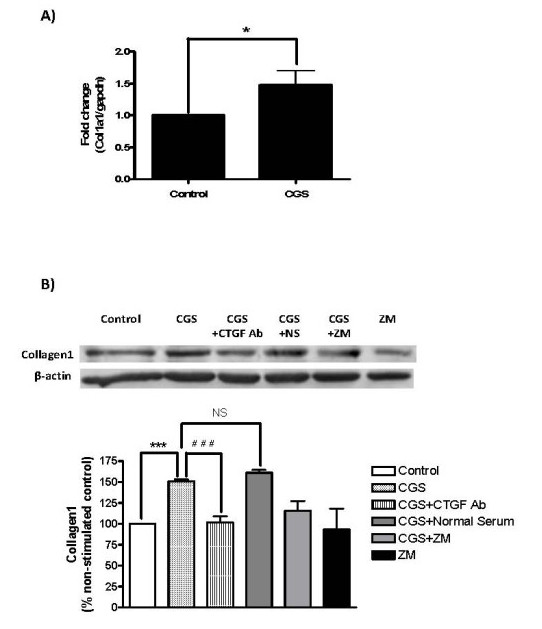
**CTGF is involved in the A_2A _receptor-mediated collagen production**. NHDF cells were incubated with CGS-26180 at 1 µM over **A) **4 h for COL1A1 mRNA analysis or **B) **during 24 h for Western-blotting after 16 h-starvation. Where indicated, the neutralizing antibody to CTGF (Ab; 1:250) or the A_2A_R antagonist ZM241385 (10 µM) was added prior to incubation with CGS-21680. Bands were quantified and data represent means ± S.E.M. from three to six independent experiments. Statistics were performed by one-way ANOVA with post-hoc analysis by Newman-Keuls test, ***P <0.001 CGS-26180 *vs*. non-stimulated control; ^###^*P *<0.001 CGS-26180 *vs*. CGS-26180 + CTGF Ab; and NS = non-significant, CGS-26180 *vs*. CGS-26180 + Normal Serum.

We have previously shown that adenosine A_2A _receptor occupancy promotes collagen type I production at both the message and protein levels [6]. Since A_2A _receptor occupancy also enhanced CTGF secretion, we determined whether CTGF is a prerequisite for A_2A _receptor-mediated collagen I production. We first investigated the mRNA levels for COL1A1 (Figure 4A), and found a significant increase following incubation with CGS-21680 (1.47 ± 0.23-fold of control, *n *= 6, *P *<0.05). We therefore sought to analyze the protein levels of collagen I and, as previously demonstrated [6,12], we found that CGS-21680 treatment increased collagen I protein by 50.1 ± 2.3% compared with control (*n *= 3, *P *<0.001) (Figure 4B). However, the addition of a neutralizing antibody to CTGF completely abrogated the CGS-21680-induced increase in collagen I protein (101.7 ± 7.6% of control, *n *= 3, *P *<0.001 vs.CGS), but control antiserum did not diminish the CGS21680-induced increase (161.2 ± 3.3% of control, *n *= 3, *P *= NS). These results suggest that increased secretion of CTGF is involved in the A_2A _receptor-mediated collagen I production.

### A_2A_R activation prevents TGF-β1 release

**Figure 5:**
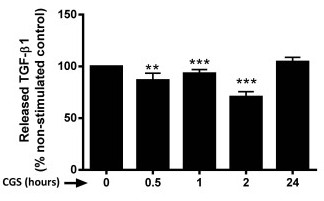
**TGF-β1 secretion is decreased by A_2A_R activation at earlier time points and restored after 24 hours**. NHDF cells were serum-starved and incubated with CGS-26180 1 µM at the indicated time periods and released TGF-β1 levels were analyzed by ELISA. Data represent means ± S.E.M. from four independent experiments. Statistics were performed by unpaired-t test, ***P <0.001, **P <0.01, *vs*. non-stimulated control.

It has been previously described that A_2A_R stimulation increases the profibrogenic factor TGF-β1 at the mRNA level in T cells [24]. Similarly, A_2A_R-deficient mice exhibit highly decreased levels of TGF-β [25]. Since it is well- known that TGF-β1 down-regulation of Fli1 promotes fibrogenesis [20,26-28], to rule out the possibility that the Fli1/ CTGF/ collagen pathway was induced via up-regulation of TGF-β1 following CGS21680 incubation, we studied TGF-β1 release after A_2A_R activation. As shown in Figure 5, released TGF-β1 was significantly reduced following two-hour incubation with the A_2A_R agonist, CGS21680. After 24 h, released TGF-β1 levels were restored.

## Discussion

We have previously shown that adenosine promotes fibrogenesis in the skin *in vivo *[9], that A_2A_R blockade prevents scarring *in vivo *[5] and activation of the adenosine A_2A _receptor but not A_1 _or A_2B _receptors promotes dermal extracellular matrix protein production including type I collagen in primary human dermal fibroblasts, and in a murine model of scleroderma [6]. Others have shown that adenosine A_2A _receptor ligation increases collagen production and induces a pro-fibrotic phenotype in dermal fibroblasts isolated from scleroderma patients, in part by activation of the CB1 cannabinoid receptor [11].The present study strongly suggests, for the first time to our knowledge that an important mechanism for the adenosine-mediated promotion of fibrogenesis occurs at least in part through the regulation of the Ets factor, Fli1, and connective tissue growth factor (CTGF; CCN2). The importance of Fli1 in the regulatory mechanism involved in the transcriptional control of collagen gene expression has been well-described. Characterization of the COL1A2 promoter has identified a critical Ets-binding site mediating Fli1 inhibition [15]. In fact, Fli1 has been previously described to function as a key regulator of the collagen homeostasis in the skin *in vivo *by repressing the collagen genes COL1A1, COL1A2, COL3A1, COL5A1 and COL5A2 [21] and has also been shown to be significantly reduced in clinically involved skin of patients with scleroderma [16]. Other studies have shown that inhibition of Fli1 by TGF-β and the subsequent displacement of Fli1 from the CTGF promoter results in the up-regulation of CTGF and the COL1A1 and COL1A2 genes in human dermal fibroblasts, strongly suggesting that TGF-β-mediated Fli1 suppression is involved in the activation of the profibrogenic gene program in fibroblasts [19]. In this regard, the secreted protein CTGF [29,30] is a major mediator of the profibrotic effects of TGF-β [19] and an important mediator of fibrosis since its constitutive expression in fibroblasts is sufficient to promote fibrotic disease *in vivo *[31].

Transforming growth factor (TGF-β) is known to be a critical regulator of matrix synthesis [26-28], and Fli1 is phosphorylated and acetylated following TGF-β1 treatment in dermal fibroblasts [21,26-28]. On the other hand, acetylation of Fli1 by TGF-β1 renders the Fli1 protein less stable, providing an important mechanism for the pro-fibrotic actions of TGF-β1 [20,26-28]. Previous reports suggest a link between dysregulation of Fli1 protein and scleroderma. Fli1 protein is markedly down-regulated in lesional fibroblasts from scleroderma patients [16]. Epigenetic mechanisms may also be responsible, in part, for the repression of Fli1 gene in scleroderma *in vivo *and increased methylation of the Fli1 promoter region has been demonstrated in SSc fibroblasts and skin biopsies from SSc patients [32]. Our prior work indicates that adenosine and the A_2A _receptor play a role in fibrosis in models of scleroderma. Mice lacking the A_2A _receptor are protected from development of dermal fibrosis following bleomycin exposure and A_2A _receptor activation promotes collagen type I and III production [12]. We, therefore, sought to analyze if the A_2A _receptor regulates collagen production via Fli1 and CTGF pathways.

Our present studies have shown that adenosine A_2A _receptor activation in human dermal fibroblasts with the agonist CGS-21680 promotes a reduction of Fli1 mRNA and protein expression in the nucleus, as well as increases in CTGF mRNA and protein expression and secretion. These findings are consistent with previous observations that Fli1 directly inhibits CTGF expression, and knockdown of Fli1 dramatically up-regulates CTGF in dermal fibroblasts [19]. Furthermore A_2A _receptor activation promotes an increase in collagen type I production which is prevented by CTGF neutralization (Figure 4B). This strongly suggests that Fli1 down-regulation and CTGF up-regulation precede collagen induction upon A_2A_R activation. Interestingly, release of TGF-β1 was reduced by A_2A_R activation. Our results therefore raise the possibility that A_2A_R and TGF-β1 may prevent excessive fibrosis by compensating its redundant pro-fibrogenic effects.

Recently, it has been suggested that hypoxia promotes fibrogenesis [33,34]. In particular, hypoxia up-regulates CTGF expression through activation of HIF-1α in dermal fibroblasts from scleroderma patients, and thereby contributes to the progression of skin fibrosis [35]. In this respect, as a critical mediator during hypoxia, the actions of adenosine are potentiated by HIF, which has been shown to stimulate the production of extracellular adenosine [36,37], and suppresses both adenosine uptake into the intracellular compartment and its intracellular metabolism [36,38,39] (reviewed in [40]). In fact, although HIF-1α has been shown to up-regulate A_2B_R during conditions of hypoxia [39] suggesting a role for the A_2B_R in tissue protection [41][42], the A_2A_R may likely contribute to the role of adenosine in ischemic settings [39,42]. Therefore, further studies will be needed to fully understand the interplay between hypoxia, HIF-1α and adenosine in skin fibrosis.

**Figure 6:**
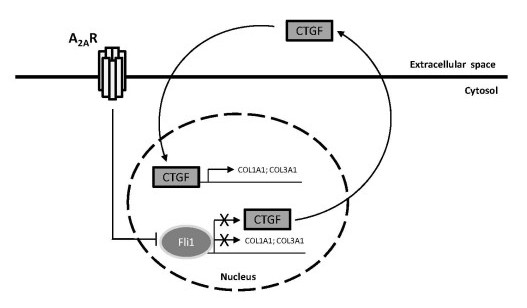
**Schema depicting the contribution of the Fli1 and CTGF pathways on the A_2A _receptor-mediated increase in collagen production**.

A schema for the involvement of the described pathways in promoting the profibrotic effects of A_2A _receptor activation is provided in Figure 6. Taken together, these data suggest that the A_2A _receptor-mediated increase in collagen type I is, at least in part, mediated by Fli1 down-regulation and therefore CTGF and collagen gene derepression. Thus, the adenosine A_2A _receptor-mediated loss of inhibition of dermal matrix protein synthesis through regulation of Fli1, and the resultant increase in the production of matrix and profibrogenic growth factors such as CTGF, is likely a contributor to the pathogenesis of skin thickening in scleroderma.

## Conclusions

In summary, the results of our present work confirm previous observations of the role of the adenosine A_2A _receptor in the pathogenesis of dermal fibrosis in conditions such as scleroderma by promoting the production of tissue matrix and profibrotic mediators. These studies suggest new mechanisms for the profibrogenic effects of the A_2A _receptor by suppressing Fli1 expression and increasing CTGF secretion. Such mechanisms may play a role in matrix production in other fibrosing diseases, such as cirrhosis, and these mechanistic targets may be further explored as potential anti-fibrotic therapies.

## Abbreviations

A_2A_R: Adenosine A2A receptor; Col1: collagen type I; Fli1: Friend leukemia integration 1 transcription factor; CTGF: connective tissue growth factor. HIF: Hypoxia-Inducible Factor.

## Competing interests

EC and BC hold a patent on the use of adenosine A_2A _receptor antagonists to inhibit fibrosis. All other authors declare no competing interests.

## Authors' contributions

EC and BC participated in study design and coordination, participated in data interpretation and manuscript preparation. EC and PF participated in the statistical analysis. HL, PF, AL and MP-A participated in sample acquisition and data interpretation. AB and MT participated in study design and coordination. All authors read and approved the final manuscript.
